# Can exercise attenuate the negative effects of long COVID syndrome on brain health?

**DOI:** 10.3389/fimmu.2022.986950

**Published:** 2022-09-16

**Authors:** Wei-Peng Teo, Alicia M. Goodwill

**Affiliations:** Physical Education and Sports Science Academic Group, National Institute of Education, Nanyang Technological University, Singapore, Singapore

**Keywords:** physical exercise (EX), cognition, COVID-19, neuroinflammation, brain-derived growth factor (BDNF)

## Abstract

The impetus for many governments globally to treat the novel coronavirus (COVID-19) as an endemic warrant more research into the prevention, and management of long COVID syndrome (LCS). Whilst the data on LCS remains scarce, reports suggest a large proportion of recovered individuals will experience ongoing neuropsychological symptoms, even with mild disease severity. The pathophysiology underlying LCS is multifaceted. Evidence suggests that altered inflammatory, neurotrophic, and neurotransmitter pathways within the brain contribute to neuropsychological symptoms reported following COVID-19. Exercise or regular physical activity has long been shown to have positive effects on brain health and cognition through exerting positive effects on inflammatory markers, neurotransmitters, and neurotropic factors analogous to the neurophysiological pathways proposed to be disrupted by COVID-19 infection. Thus, exercise may serve as an important lifestyle behavior in the management of LCS. In this opinion article, we present the evidence to support the positive role of exercise in the management of cognitive symptom that manifest with LCS and discuss important considerations and interactions with cardiorespiratory and exercise tolerance complications that often present for individuals experiencing LCS. We highlight the need for more research and training of sports medicine practitioners and clinical exercise physiologists in the management of LCS with exercise and call for further research to understand the optimal dose-responses and exercise prescription guidelines for cognitive benefits and minimizing other complications.

## Introduction

The novel coronavirus (COVID-19) pandemic has affected the lives of millions as it disrupts economies, businesses, and daily activities of people around the world. As of the 16^th^ August 2022, the World Health Organization reported an estimated 590,659,276 global confirmed cases, while 6,440,163 confirmed deaths have been reported (1). The symptoms of COVID-19 have been well-described, which includes but not limited to, fever, upper-respiratory tract infection, fatigue, sore throat, loss of taste and smell and cough being the most common. While most patients recover from COVID-19, emerging evidence suggests that more than half of recovered COVID-19 patients, including those with mild symptoms, show long-lasting post-COVID symptoms, referred to as long COVID syndrome (LCS). This includes reduced general well-being (e.g., weight loss and fatigue), mobility, mental health (e.g., increased feelings of anxiety), cardiovascular, cardiorespiratory and cognitive functioning (2).

Unlike other variants from the coronavirus family, COVID-19 has the unique ability to affect multiple organs, including the central nervous system (CNS). Indeed, studies have reported that up to 80% of COVID-19 patients can suffer from a host of neurological and neuropsychiatric complications such as loss of smell, and noticeable declines in executive functioning (i.e., working memory, attention) and feeling of fatigue or brain fog even months after recovery (3). While the mechanisms for how COVID-19 affects the CNS remain complex and unclear, preliminary findings from autopsy reports (4) and neuroimaging studies (5, 6) have shown structural and functional changes within the brain that may be associated with LCS-related neurological impairments. However, to date, treatments to attenuate neurological and cognitive symptoms of LCS remain limited.

One potential avenue to remediate cognitive symptoms of LCS is with exercise. Now that most countries are declaring COVID-19 as an endemic, and the number of vaccinations is increasing, emphasis on interventions to reduce symptom severity, duration, and improve long-term outcomes should be encouraged. Certainly, organizations such as the American College of Sport Medicine (ACSM) are pushing for a return to physical activity, and in particular, safe return to exercise following COVID-19 infection (7). It is well-documented that performing exercise on a regular basis confers benefits to a host of bodily systems including the CNS (8–10). Indeed, recent large-scale studies provide evidence for the protective effects of regular physical activity, particularly physical activities performed at moderate-to-vigorous intensities, against severe complications and death from COVID-19 (11). Similarly, physical inactivity has also been associated with higher risk of more severe COVID-19-related outcomes (12). It is therefore conceivable that exercise may also be used to rehabilitate and attenuate LCS neurological complications, such as declines in cognitive functioning. However, there are barriers for clinicians and exercise practitioners to consider when implementing physical exercise following recovery from COVID-19. For example, sequalae of COVID-19 may include cardiac damage and respiratory complications, which is likely to increase the risk of adverse events or exercise intolerance following COVID-19. Further, as the evidence for exercise on cognitive impairment in LCS remains limited, is it unclear as to what the limits of exercise prescription should be, to derive any cognitive benefits. These uncertainties may therefore hinder the prescription of exercise as a first approach. Thus, there is a need to evaluate our understanding of how exercise benefits CNS function and how this impacts the safe return to exercise following COVID-19 infection.

In this current review, we aim to discuss the potential role of exercise to attenuate the effects of LCS on cognitive functioning. We will highlight the existing knowledge on the underpinning mechanisms for cognitive impairments such as difficulties in concentration/attention, memory and executive functioning and brain fog that have been reported with LCS and suggest potential mechanisms in which both aerobic and resistance forms of exercise may be used to attenuate these cognitive symptoms and optimize overall brain health following COVID-19. As countries work relentlessly to manage and contain the aftereffects of COVID-19, many individuals will undoubtedly be affected by symptoms of LCS. Physical exercise may therefore represent a viable, cost-efficient, and safe way to improve the lives of COVID-19 survivors.

## Effects of COVID-19 on the brain

To date, there appears to be no clear link between autopsy findings at the structural level of the brain as most patients that succumb to COVID-19 have a high prevalence of other comorbidities (i.e., neurological diseases, neurodegeneration, prior strokes and tumors and atherosclerosis). A previous mini systematic review by Mukerji and colleague (4) suggested that more than half (92 out of 142 autopsies, 65%) of autopsy findings show no significant or acute abnormalities in gross brain structures associated with COVID-19 infections. Out of the remaining 35% (50 out of 142 autopsies), the most common abnormalities identified were signs of brain hemorrhaging, acute/subacute ischemic brain injury, and oedema. Further, as the characteristics of patients that succumbed to COVID-19 in this study were diverse (i.e., age, medical history, severity of symptoms), drawing any conclusive associations between structural brain changes and COVID-19 symptoms is limited.

While brain autopsies provide acute evidence of COVID-19 to affect the brain, it still does not explain the cognitive and neurological symptoms observed with milder cases, especially those suffering from LCS. Recent neuroimaging evidence has provided support for structural, functional, and cognitive abnormalities associated with even with mild COVID-19 cases. For example, a case report published in the Journal of Neurology by Hugon et al. (6) suggested that brain fog may be underpinned by hypometabolism in the cingulate cortex. Using (18)F-fluorodeoxyglucose positron emission tomography (FDG-PET) scans in two confirmed LCS patients with no cardiovascular complications, the authors found hypometabolism in the posterior and anterior cingulate cortex and precuneus in one patient, while the other showed hypometabolism in only the anterior cingulate cortex, with the posterior cingulate cortex less severely affected. Both patients displayed mildly affected episodic and visuospatial memory and deficits in executive functioning. It should however be noted that the small sample size in Hugon’s study limits the interpretation of their findings. To overcome the issue of sample size, a study published in Nature by Douaud et al. (5) compared structural magnetic resonance imaging (MRI) scans obtained from the UK BioBank data between 401 COVID-19 survivors and 384 age-matched controls. What makes this study robust was the availability of pre- and post-infection structural MRI scans that helped to eliminate any influence of pre-existing conditions on brain architecture. This study showed that in COVID-19 survivors, grey matter thickness in the orbitofrontal cortex and parahippocampal gyrus was reduced, there was increased tissue damage to brain regions functionally connected to the primary olfactory cortex, and lastly, greater reduction in global brain size. These structural changes were concomitant with greater cognitive impairments in COVID-19 survivors compared to controls.

From a cellular standpoint, there is emerging evidence to suggest that COVID-19 may affect the brain *via* three main factors; 1) increased inflammatory response, 2) modulation of brain-derived neurotrophic factor (BDNF), and 3) disruption of glutaminergic pathways. In support for the first factor, several studies have now shown increased inflammatory responses within the brain and circulatory system are positively associated with the severity of COVID-19 symptoms (13–15). Moreover, studies have further reported that patients with existing cardiometabolic and/or cardiovascular symptoms (e.g., hypertension, obesity, and type II diabetes) are associated with greater risk of severe COVID-19 complications and mortality (16–18). It has been postulated that increased visceral adiposity and poor glucose control may worsen COVID-19 responses by promoting secretion of hormones and cytokines that are associated with systemic inflammation. Similarly, inflammatory responses in the brain associated with COVID-19 have been reported, albeit in small case reports (19, 20). In a study by Schwabenland et al. (21) increased neuroinflammatory markers and immune responses were concomitant with astrocytosis, axonal damage, and blood-brain barrier leakage in 25 patients that succumbed to COVID-19 compared with controls.

Apart from the role of inflammation on COVID-19 responses, brain-derived neurotrophic factor (BDNF) expression may have potential implications for severity of COVID-19 symptoms. Some studies have suggested that severity of COVID-19 symptoms was negatively associated with circulating levels of BDNF, that is, patients with moderate to severe symptoms presented with lower circulating BDNF (22, 23) while recovery from COVID-19 infection was associated with increased levels of circulating BDNF (22). As BDNF plays a key role in maintaining neuronal survival, growth, and function, this may be a plausible explanation for changes in the CNS that are associated with COVID-19 infection and LCS. It should however be noted that the directionality and mechanisms between the relationship of BDNF and COVID-19 symptoms are complex and likely to be mediated by other signaling pathways (24).

Finally, a study by Yesilkaya et al. (25) provided preliminary evidence that implicated transient dysfunction in the glutaminergic pathway of the dorsolateral prefrontal cortex (DLPFC) following recovery from COVID-19 infection. In this case study, magnetic resonance spectroscopy (MRS) was performed on a single patient one week and three months after recovery from COVID-19 infection. The authors found that N-acetylaspartate, glutamate, and glutamate/glutamine ratio was significantly reduced in the DLPFC at 1-week post-recovery that was concomitant with cognitive impairments such as poor attention and memory retrieval. The patient was subsequently advised to engage in cognitive training for three months without medication. At three-month follow-up, glutamate, and glutamate/glutamine ratio was significantly increased, with moderate increase in N-acetylaspartate.

Taken together, the mechanisms in which COVID-19 affects cognitive functioning appear to be multifaceted; underpinned by structural abnormalities, increased inflammatory responses, and impaired cellular pathways that support CNS homeostasis and functions (e.g., BDNF and glutamate). However, it should be noted that evidence from COVID-19 survivors is still sparse and that most evidence to date has been gathered mainly from autopsy or case study reports in those who had severe symptoms. Therefore, gaining a more comprehensive understanding of the effects of LCS on cognition and its underlying mechanisms will allow for the development of effective exercise strategies to mitigate the negative impact of LCS on the brain.

## The role of exercise to attenuate the negative effects of COVID-19 on the brain and cognition

It is now widely accepted that exercise, either acute or chronic in duration, can have a strong effect on brain structure and function. There is robust evidence to suggest that aerobic and resistance forms of exercise have beneficial effects on cognitive functioning across the lifespan and in neurodegenerative disease populations (8, 26–28) While there are currently no studies to date reporting on the effects of exercise on cognitive function in patients with LCS, we will draw on evidence from exercise neurophysiology studies to provide theoretical support for the use of exercise to attenuate cognitive symptoms in LCS.

Perhaps the most direct support for exercise on LCS-related cognitive impairments is its role on BDNF and cognitive functioning. Exercise have been shown to increase BDNF expression and improve cognitive functioning across the lifespan and in various clinical populations such as neurodegenerative disorders, mental disorders, and stroke. A meta-analysis conducted by Szuhany et al. (29) further demonstrated that while a single bout of exercise can increase expression of BDNF, regular exercise elicited a stronger effect on BDNF expression. What was interesting to note was that this review further showed that regular exercise had a small but significant positive effect on resting BDNF levels. It is therefore plausible that patients who performed regular exercise before COVID-19 infection may experience less COVID-related cognitive impairments compared to patients that were sedentary. This may be due to higher levels of resting BDNF, and physical fitness levels associated with regular exercise to help stave off more serious implications of COVID-19. In addition, performing aerobic or resistance exercises following recovery from COVID-19 may help to spur expression of BDNF, which may help to attenuate cognitive symptoms associated with LCS. Indeed, there is growing evidence to show that even acute aerobic (30) and resistance exercises (31, 32) or sports participation (33) at moderate to vigorous intensities can lead to improved cognitive functioning and potentially increase serum BDNF expression (34–36).

Another key aspect of exercise on CNS function is an increase cerebral blood flow (CBF), which has been shown both during and after exercise. The ability to regulate CBF is vital for neuronal survival and function, and impairments to CBF due to even mild pathology can lead to hypoperfusion of brain regions and ultimately affect cognitive functioning (37, 38). Recent neuroimaging evidence on COVID-19 patients showed that CBF is reduced in cortical and subcortical structures in patients that were severely ill, compared to those that had mild symptoms and healthy controls up to at least three months post-recovery (39, 40). While in most cases usual exercise routines are not advised to be performed during symptomatic infection, particularly if patients are suffering from fevers and chest infection, light mobility exercises such as passive and active stretching and/or gentle walking may be beneficial for improving blood circulation and CBF particularly during prolonged bedrest and home-isolation.

In support of this line of evidence, previous animal studies in rats suggest that chronic (i.e., 1-3 months) cerebral hypofusion is detrimental to cognitive functioning, however, chronic (i.e., 4-12 weeks) physical exercise at moderate intensities improved spatial working memory (41–44). These studies further highlight that the improvements in cognitive functioning were concomitant with increased cerebral angiogenesis and vascular endothelial growth factor expression (43), neuronal remyelination (41) and improve capillary function (44) within the brain. Beyond vascularization, aerobic exercise and fitness has been associated with greater levels of N-acetylaspartate (45) and regulation of glutamate levels (46) in the brain, which may be linked to improved cognitive performance.

Lastly, inflammation within the CNS due to the release of pro-inflammatory cytokines is likely to be associated with severity of COVID-19 symptoms, which researchers have dubbed a “cytokine storm”. Bouayed and Bohn (47) proposed a “Two-Hit Hypothesis” that suggest a combination of poor lifestyle and environmental factors in early life (first hit), and disease and infection later in life (second hit) may upregulate pro-inflammatory processes such as increased release of cytokines and inflammatory markers such as interleukin (IL)-6, tumor necrosis factor (TNF)-α, and interferon (IF)-γ that may explain why certain individual show worsened COVID-19 symptoms compared to others. In this sense, exercise may play a critical and direct role in attenuating the inflammatory response following COVID-19 that underpin cognitive impairments. Indeed, studies in both animal and human models have provided evidence for a reduction in pro-inflammatory cytokines and markers, which suppresses neurotrophic factors such as BDNF and IGF-1 (48). For example, a study by Belotto et al. (49) reported a significant reduction in cytokines, TNF-α and IL-1β in diabetic rats after three-weeks of moderate aerobic exercise (60% VO_2max_, 30mins/day, 6 days/week). And while it remains unclear as to how exercise may attenuate LCS *via* attenuation of pro-inflammatory pathways, a recent review by Rebello et al. (50) provides a clear mechanistic hypothesis of how exercise may potentially reduce LCS symptoms. The authors proposed that exercise-induced IL-6 release (often viewed as a pro-inflammation marker) by skeletal muscles is not preceded by the release of other pro-inflammatory markers such as TNF-α and IL-1β. Indeed, a study by Starkie et al. (51) provided evidence for the role of exercise and exogenous IL-6 infusion in attenuating endotoxin-induced TNF-α response (via administration of *Escherichia coli* lipopolysaccharide endotoxin (0.06 ng/kg) i.e., to induce low-grade inflammation). Therefore, the release of IL-6 in response to exercise creates an anti-inflammatory environment as opposed to a pro-inflammatory one.

## Current recommendations, barriers and considerations for exercise prescription following COVID-19

**Figure 1:**
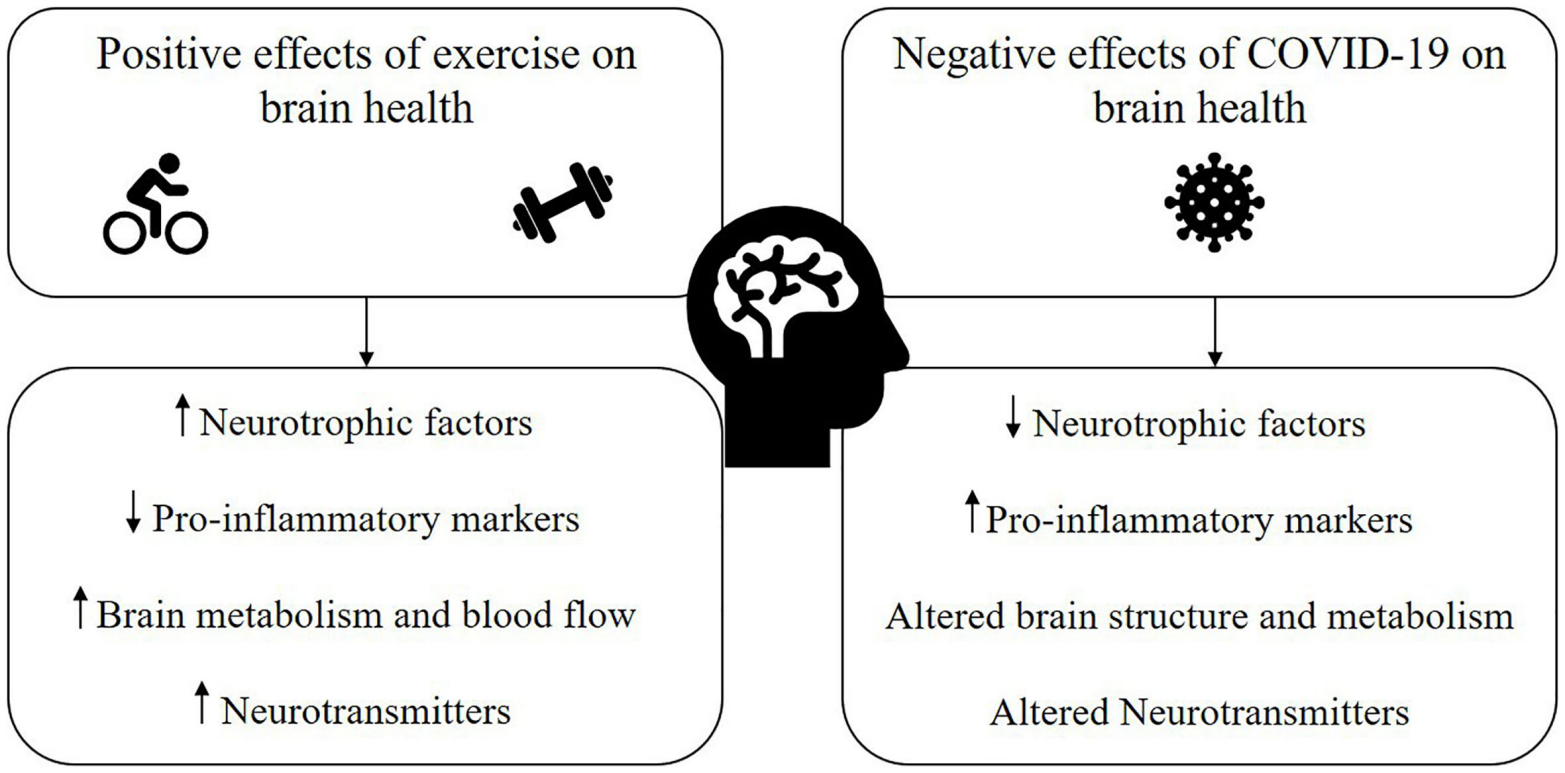
Proposed mechanisms through which exercise has the potential to mitigate the negative impact of COVID-19 and/or long COVID syndrome (LCS) on brain health.

The current evidence supporting the neuroprotective, pro-inflammatory, and cerebrovascular benefits of exercise on the brain suggests that exercise could be one promising avenue to counter the negative effects of COVID-19 on the brain and cognition (**Figure 1**). However, the interaction of exercise with potential cardiorespiratory and other complications often coinciding with LCS must be considered as part of an exercise prescription plan for individuals recovering from COVID-19. Paradoxically, whilst we argue exercise to be an important lifestyle behavior in managing cognitive symptoms of LCS, many recovered individuals have also reported exercise intolerance, which has been attributed to general chronic fatigue, myopathy, post-exertional malaise, and postural tachycardia syndrome (POTS) (52, 53). In these cases, generalized exercise programs also have the potential to exacerbate symptoms and must be carefully monitored by a team of allied health, exercise, and medical professionals. Moreover, patients experiencing shortness of breath or chest pain after recovering from initial COVID-19 infection are recommended to be periodically evaluated for signs of myocarditis during their return to sports or exercise (54, 55).

Another consideration in managing neuropsychological symptoms post-covid is the type of exercise prescription. The most recent ACSM position statement on return to exercise post-COVID proposed return-to-exercise guidelines for several risk categories based on age, co-morbidities, initial infection severity, and persistent symptoms (7). In general, the guidance suggests increasing stamina by commencing with activities of daily living, light aerobic exercise (e.g., walking), and body weight resistance training to repair muscle damage and strength. It is recommended these exercises be performed gradually from 7-days post-symptoms in the absence of symptom exacerbation, resurgence, or post-exertional malaise. Hughes et al. (56) echoed this approach, emphasizing symptom severity and duration as a guide for return to exercise post-COVID-19 infection. The authors proposed exercise to be re-introduced following symptom improvement/resolution at 50% of one’s regular exercise routine and intensity, with ongoing monitoring of exercise-induced fatigue or intolerance. This highlights the importance of ongoing symptom monitoring and exercise tolerance for those experiencing symptoms of LCS, and these individuals would benefit from referral to Clinical Exercise Physiologists to work closely with allied health and medical practitioners on progressive return to exercise plans. A return to physical activity framework has also been proposed by Salman et al. (57), which suggested a starting exercise intensity of 6-8 points on the rating of perceived exertion (RPE) scale, and an increase of 2-3 points every 7 days until a return to regular exercise pattern is established with an RPE score of 15 points that is well-tolerated.

Currently, much of the current return-to-exercise guidelines are based off theoretical knowledge, studies in hospitalized patients undergoing rehabilitation, or athletes (58). Thus, there is a need for more research into the optimal and appropriate exercise prescription for management of cognitive and other neuropsychological symptoms within the general population with LCS, particularly in mild COVID-19 patients who have undergone home recovery. Of the intervention studies conducted to date, a review of six studies in patients with LCS showed improvements in immune markers with no symptoms of exhaustion during aerobic cycling or walking exercise at intensities between 60-80% of their heart-rate maximum for 20-60 min and at 2-3 times per week (59). Further, a review of resistance-based exercise training performed at a low volume (e.g., between 1-2 sets, 8-10 repetitions, 30-80% of their 1 repetition maximum [1RM]) also improved functional capacity and quality of life in recovered patients (60). Providing further support for the incorporation of resistance exercise into post COVID-19 recovery, low intensity (30-60% of 1RM, 8-20 repetitions, 30-60 sec rest between exercises) resistance exercise has long been safely used in individuals with acute and chronic respiratory conditions and cardiac rehabilitation (61), and induces structural and functional brain changes that may underpin improvements in cognition (62). In this regard, future research would benefit from understanding the role of resistance training combined with mobility and aerobic activities in mitigating cognitive deficits following COVID-19, whilst minimizing cardiorespiratory complications or symptom exacerbation.

## Conclusion

It is without a doubt that the COVID-19 pandemic has disrupted many aspects of life. During the initial phase of managing the spread of COVID-19, social and physical isolation were enforced in many countries which led to a decrease in the level of physical activity, fitness and quality of life worldwide (63–65). Even after being declared an endemic, the long-term effects of COVID-19 are still apparent with careful and systematic considerations for the safe return to exercise and sports being implemented worldwide. For individuals that have recovered from COVID-19, symptoms of LCS such as cognitive deficits remain a serious issue that may impact work performance and quality of life. To mitigate symptoms of LCS, exercise offers a cost-effective and viable solution to improve neuropsychological functioning. While exercise may offer a suitable strategy to attenuate cognitive deficits in LCS, careful prescription and management of exercise is necessary. Longer-term strategies will be necessary to implement exercise programs following COVID-19 to ensure individuals recover optimally and safely back into regular physical activity and sport.

## Author contributions

Both authors contributed equally to the conceptualization and writing of the manuscript.

## Funding

The authors would like to acknowledge the Physical Education and Sports Science Academic Group for funding received for this manuscript.

## Conflict of interest

The authors declare that the research was conducted in the absence of any commercial or financial relationships that could be construed as a potential conflict of interest.

## Publisher’s note

All claims expressed in this article are solely those of the authors and do not necessarily represent those of their affiliated organizations, or those of the publisher, the editors and the reviewers. Any product that may be evaluated in this article, or claim that may be made by its manufacturer, is not guaranteed or endorsed by the publisher.
